# Add-on implantation of the smaller-incision new generation implantable miniature telescope (SING IMT™) in pseudophakic eyes

**DOI:** 10.1186/s40942-025-00797-9

**Published:** 2026-01-23

**Authors:** Marco Pellegrini, Ginevra Giovanna Adamo, Antonio Cartabellotta, Pietro Maria Talli, Sabrina Crisafulli, Francesco Parmeggiani, Marco Mura

**Affiliations:** 1 Department of Translational Medicine, University of Ferrara, Ferrara, Italy; 2 Sant’Anna University Hospital, Via A. Moro 8, Ferrara, Cona 44124 Italy; 3 King Khaled Eye Specialist Hospital, Riyadh, Saudi Arabia

**Keywords:** SING IMT, Implantable miniature telescope, Age-related macular degeneration, Visual rehabilitation

## Abstract

**Background:**

To describe a novel surgical technique for add-on sulcus implantation of the Smaller-Incision New Generation Implantable Miniature Telescope (SING IMT™) in pseudophakic eyes with end-stage age-related macular degeneration (AMD).

**Methods:**

Two pseudophakic patients with bilateral end-stage AMD underwent add-on SING IMT™ implantation in the ciliary sulcus. Pre- and postoperative evaluations included best-corrected distance visual acuity (BCDVA), anterior segment OCT (AS-OCT), corneal endothelial cell counts, and subjective visual function assessments, with follow-up to 6 months.

**Results:**

Both surgeries were uneventful. The devices remained stable and well-centered with no significant inflammation, corneal decompensation, or intraocular pressure elevation. BCDVA improved by 3–4 ETDRS lines, and both patients reported improved daily visual function. Corneal endothelial loss was < 5% at 6 months.

**Conclusions:**

Add-on sulcus implantation of the SING IMT™ in pseudophakic eyes is surgically feasible and well tolerated, expanding the potential indications of this visual rehabilitation device to a broader AMD population.

**Supplementary Information:**

The online version contains supplementary material available at 10.1186/s40942-025-00797-9.

## Background

Age-related macular degeneration (AMD) remains the leading cause of irreversible central vision loss in older adults worldwide, profoundly affecting patients’ quality of life and independence [1]. While advances in pharmacologic therapy have significantly improved outcomes in neovascular AMD, there are currently no effective treatments to restore central vision in patients with end-stage or atrophic AMD. For this group, visual rehabilitation relies on optical or surgical strategies designed to project images onto healthier peripheral retina, thereby improving functional vision.

The Implantable Miniature Telescope (IMT™; VisionCare, Inc.) was developed as an intraocular device to provide magnified retinal images, improving central visual function in patients with bilateral, end-stage AMD. Clinical trials of the first-generation IMT demonstrated meaningful improvements in visual acuity and vision-related quality of life; however, the original device required a relatively large corneal incision (10–12 mm), which limited its adoption due to the risk of surgically induced astigmatism and longer recovery times [2–4].

The Smaller-Incision New-Generation Implantable Miniature Telescope (SING IMT™) represents an evolution of this technology, incorporating optical and surgical design improvements that allow implantation through a reduced incision size (~ 8 mm) while maintaining optical performance. The SING IMT™ aims to reduce intraoperative trauma, preserve corneal integrity, and simplify the surgical procedure [5–7].

An important limitation of the SING IMT™ is that implantation currently requires the patient to be phakic, as the device is designed to replace the crystalline lens. Consequently, individuals who have previously undergone cataract extraction with intraocular lens (IOL) implantation are not eligible for the procedure. This restriction significantly narrows the eligible patient population, as the majority of patients with advanced AMD have already undergone cataract surgery [8]. Therefore, expanding the applicability of this technology to pseudophakic patients would be important to broaden the clinical utility of the device.

The present study aims to describe a novel surgical technique for add-on sulcus implantation of the SING IMT™ in two pseudophakic eyes, demonstrating the feasibility of this approach in previously ineligible patients.

## Methods

This prospective interventional case report describes a novel surgical technique for add-on sulcus implantation of the SING IMT™ (VisionCare, Inc.) in two pseudophakic eyes with end-stage AMD. Both surgeries were performed at the Sant’Anna University Hospital, University of Ferrara, Italy. The study was conducted in accordance with the tenets of the Declaration of Helsinki, and informed consent was obtained from both patients after detailed explanation of the off-label nature of the procedure and its potential risks and benefits.

Two pseudophakic patients with bilateral, end-stage AMD and severe central vision loss were included. Both had previously undergone uncomplicated cataract surgery with posterior chamber monofocal intraocular lens (IOL) implantation and demonstrated stable ocular status, adequate corneal clarity, and sufficient anterior chamber depth on slit-lamp and optical biometry evaluation. Exclusion criteria included active ocular inflammation, corneal endothelial compromise, uncontrolled glaucoma, or other retinal pathology limiting visual potential.

Baseline evaluations included distance and near best-corrected visual acuity (BCVA), slit-lamp biomicroscopy, dilated fundus examination, intraocular pressure (IOP) measurement, specular microscopy, anterior segment and macular optical coherence tomography (OCT).

All procedures were performed by the same experienced anterior segment surgeon under sub-tenon anesthesia. The surgical video is available in Supplementary Material 1. Following superior conjunctival peritomy, a superior self-sealing corneal incision of 8 mm was created. The anterior chamber was filled with a cohesive ophthalmic viscoelastic device to protect the corneal endothelium. The SING IMT™ was carefully introduced into the anterior chamber using a custom injector and positioned in the ciliary sulcus, anterior to the existing posterior chamber IOL. Proper centration and stability of the device were confirmed intraoperatively. An iridectomy was performed at the 12 o’clock position. Then, the corneal incision and conjunctiva were closed with interrupted 8 − 0 vicryl sutures.

Postoperative treatment included topical antibiotics and corticosteroids tapered over four weeks. Patients were examined on postoperative days 1 and 7, and at 1, 3, and 6 months. Follow-up evaluations included distance and near BCVA, IOP measurement, specular microscopy, slit lamp and fundus examination, and anterior segment OCT to assess proper device positioning.

## Results

**Fig. 1:**
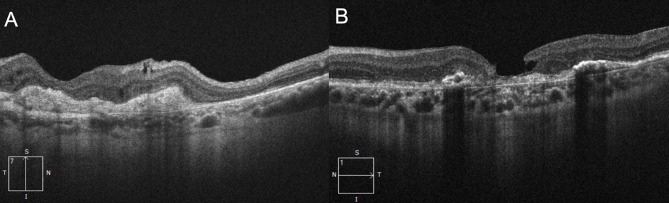
Preoperative macular OCT in patient 1 (**A**) and patient 2 (**B**)

Two female patients of 80 (patient 1) and 79 years (patient 2), with bilateral end-stage AMD with disciform scarring were enrolled (Fig. 1).

Both patients had previously undergone cataract surgery with IOL implantation in both eyes. The eye with the worst BCVA was selected for enrollment. In particular, BCVA was 19 ETDRS letters at 2 m in patient 1 and 7 ETDRS letters at 2 m in patient 2. Preoperative anterior chamber depth (ACD) had to be ≥ 3.50 mm. Specifically, ACD was 4.07 mm in patient 1 and 3.57 mm in patient 2.

Both patients successfully underwent add-on sulcus implantation of the SING IMT™ without any intraoperative complication. Mild transient anterior chamber reaction was observed in both cases at postoperative day 1, resolving within one week with topical corticosteroids.

**Fig. 2:**
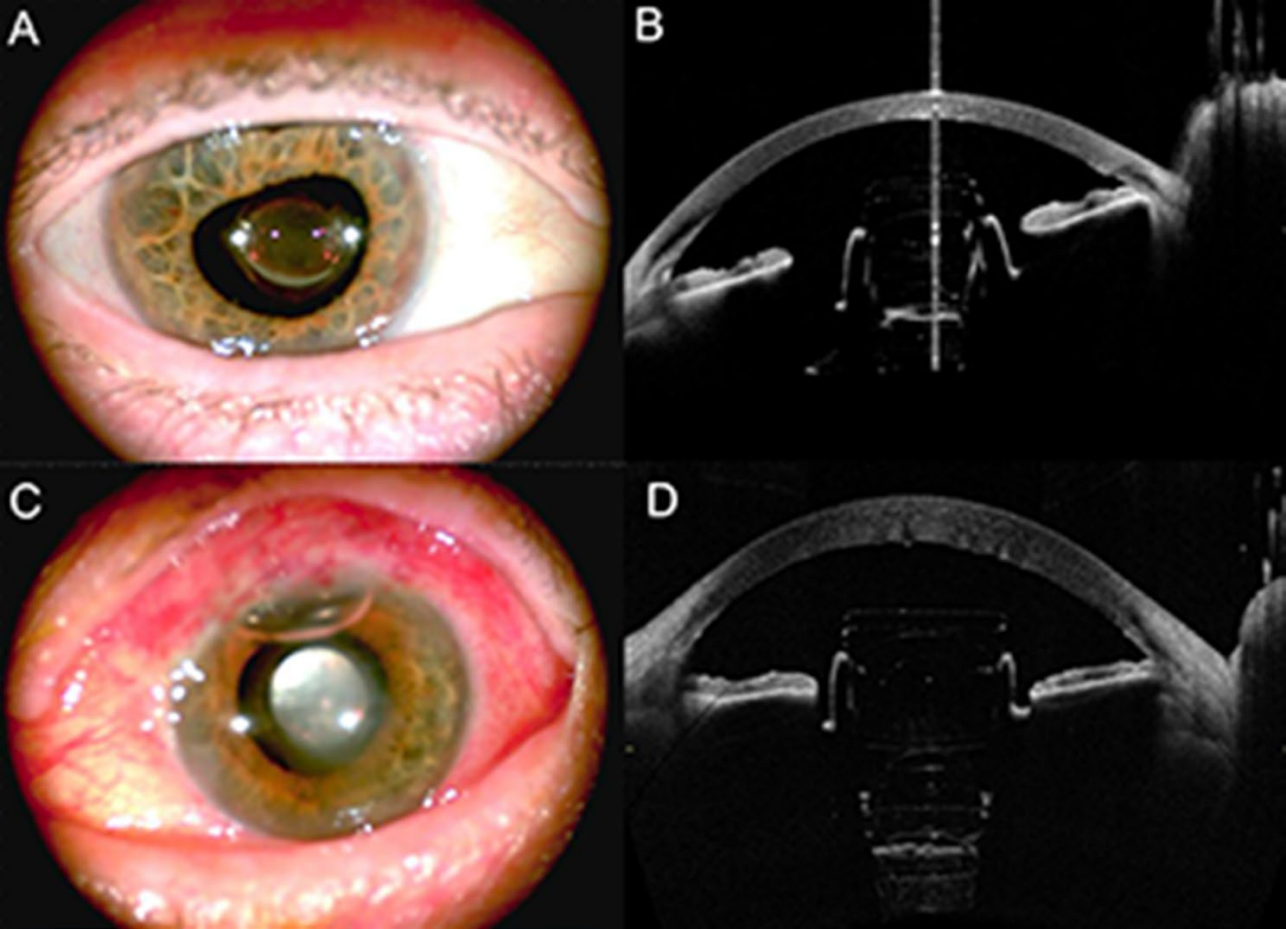
Postoperative AS photo and AS-OCT of patient 1 (**A**, **B**) and patient 2 (**C**, **D**)

As confirmed by AS-OCT imaging (Fig. 2), the devices were well centered within the ciliary sulcus at a safe distance from the corneal endothelium. 

There were no postoperative complications, such as significant inflammation, persistent corneal edema, elevated intraocular pressure, or device-related adverse events. Corneal endothelial cell counts remained stable, with less than 5% reduction from baseline values at six months (endothelial cell loss: 59 cells/mm^2^ in patient 1 and 74 cells/mm^2^ in patient 2). Fundus examination and spectral-domain OCT showed no signs of retinal edema or progression of atrophy.

At six months postoperatively, both eyes demonstrated meaningful improvement in central visual function compared with baseline. In particular, distance BCVA improved from 19 to 22 ETDRS letters at 2 m in patient 1 and from 7 to 14 ETDRS letters at 2 m in patient 2, while near BCVA measured with MNREAD™ charts at 40 cm improved from 20/200 to 20/125 Snellen in patient 1 and from 20/250 to 20/200 Snellen in patient 2. Both patients reported enhanced ability to recognize faces, read large print, and perform daily visual tasks.

Distances between the implant and the corneal endothelium at 6 months postoperatively were 1.35 mm for patient 1 and 1.68 mm for patient 2.

## Discussion

This case report describes the first successful add-on sulcus implantation of the Smaller-Incision New Generation Implantable Miniature Telescope (SING IMT™) in pseudophakic eyes with end-stage AMD. The results demonstrate that the modified surgical approach is technically feasible, well tolerated, and capable of producing meaningful improvements in visual acuity and functional vision without compromising the safety of the procedure.

The original and second-generation IMT devices were designed for implantation in phakic eyes as a replacement for the crystalline lens, offering magnification of central images projected onto healthier parafoveal retina [2–7]. However, this design inherently excluded pseudophakic individuals—who represent the vast majority of patients with advanced AMD—thereby limiting the overall applicability of the technology.

Our group previously reported implantation of the SING IMT™ in a pseudophakic eye. In that case, the IOL was explanted resulting in capsular bag damage which was managed via scleral fixation of the device through 3 Gore-Tex sutures [8]. Similarly, Savastano et al. reported IOL removal and SING IMT™ implantation in 3 pseudophakic eyes, with dislocation of the device into the vitreous chamber requiring scleral fixation in one of them [9]. The ability to perform add-on implantation of the SING IMT™ in the ciliary sulcus, anterior to an existing posterior chamber IOL, represents a meaningful step toward expanding the candidate population for this form of visual rehabilitation.

In both cases presented, the SING IMT™ was implanted through an 8-mm corneal incision and positioned securely in the sulcus without intraoperative or postoperative complications. The reduced incision size compared with earlier IMT models likely contributed to the absence of corneal edema and the preservation of endothelial cell density, with less than 5% cell loss at six months. This finding aligns with previous reports demonstrating that the smaller-incision design of the SING IMT™ reduces surgical trauma while maintaining high optical quality and field of view [5–7].

Postoperative visual outcomes in these pseudophakic eyes were comparable to those achieved with standard phakic SING IMT™ implantation. Both patients experienced improvements in BCDVA, accompanied by enhanced near vision and functional performance in daily activities such as face recognition and reading. These gains are consistent with prior clinical trial data for the IMT device family, which reported similar visual improvements and patient satisfaction in appropriately selected candidates [5–7].

Importantly, no device-related complications such as pigment dispersion, chronic inflammation, defects in the zonules, device dislocation, pupillary block, or mechanical contact with the IOL optic were observed during six months of follow-up. This suggests that, with careful patient selection and intraoperative centration, the anatomical configuration of the sulcus can accommodate the SING IMT™ without undue crowding of the anterior chamber angle or contact with corneal endothelium. Specifically, endothelium-implant distance remained the same at 6 months follow up. Nevertheless, long-term follow-up in a larger series is required to confirm the stability and biocompatibility of this add-on configuration, particularly regarding potential pigment epithelial changes, IOP elevation, or late-onset inflammatory reactions.

From a clinical perspective, this modified surgical technique broadens the scope of patients eligible for telescope-based visual rehabilitation. As most individuals with advanced AMD are already pseudophakic, adapting the SING IMT™ for secondary implantation could markedly increase accessibility to this vision-restoring technology. It is also worth noting that in a certain number of patients, explantation of the SING IMT™ device may eventually be required due to unexplained blurred or hazy vision leading to postoperative dissatisfaction [6]. In such cases, the add-on sulcus implantation approach offers a significant safety advantage, as it preserves the native capsular bag–IOL complex and allows for a safer and more straightforward device removal if necessary, reducing the need for additional intraocular surgery.

Limitations of this report include the small sample size and short follow-up duration. Additional prospective studies with longer observation periods are warranted to evaluate visual outcomes, optical interactions between the SING IMT™ and the existing IOL, and the long-term safety profile of sulcus implantation.

## Conclusions

In summary, add-on sulcus implantation of the SING IMT™ in pseudophakic eyes appears to be a feasible and safe modification of the standard surgical approach, providing meaningful visual improvement in patients previously deemed ineligible for the procedure. With further validation, this technique may expand the therapeutic options available for visual rehabilitation in end-stage AMD.

## Supplementary Information

Below is the link to the electronic supplementary material.

Supplementary Material 1

## Data Availability

The datasets used and/or analyzed during the current study are available from the corresponding author upon reasonable request.
